# Leprosy detection rate in patients under immunosuppression for the treatment of dermatological, rheumatological, and gastroenterological diseases: a systematic review of the literature and meta-analysis

**DOI:** 10.1186/s12879-021-06041-7

**Published:** 2021-04-13

**Authors:** Daniel Holanda Barroso, Jurema Guerrieri Brandão, Elaine Silva Nascimento Andrade, Ana Clara Banhatto Correia, Danielle Costa Aquino, Ana Carolina Rios Chen, Sebastian Vernal, Wildo Navegantes de Araújo, Lícia Maria Henrique da Mota, Raimunda Nonata Ribeiro Sampaio, Patrícia Shu Kurizky, Ciro Martins Gomes

**Affiliations:** 1 Programa de Pós-Graduação em Ciências Médicas, Faculdade de Medicina, Universidade de Brasília – UnB, Campus Universitário Darcy Ribeiro, Brasília, DF CEP 70910-900 Brazil; 2 Departamento de Doenças de Condições Crônicas e Infecções Sexualmente Transmissíveis – DCCI, Coordenação Geral de Vigilância das Doenças em Eliminação – CGDE, Secretaria de Vigilância em Saúde, Ministério da Saúde, Brasília, Brazil; 3 Programa de Pós-Graduação em Saúde Coletiva, Faculdade de Medicina, Universidade de Brasília – UnB, Brasília, Brazil; 4 Faculdade de Medicina de Petrópolis, Petrópolis, Brazil; 5 Faculdade de Medicina, Universidade de Brasília – UnB, Brasília, Brazil; 6 Departamento de Moléstias Infecciosas e Parasitárias, Faculdade de Medicina, Universidade de São Paulo, São Paulo, Brazil; 7 Departamento de Clínica Médica, Divisão de Dermatologia, Faculdade de Medicina de Ribeirão Preto, Universidade de São Paulo, Ribeirão Preto, SP Brazil; 8 Programa de Pós-Graduação em Medicina Tropical, Núcleo de Medicina Tropical, Universidade de Brasília – UnB, Brasília, Brazil; 9 National Institute for Science and Technology for Health Technology Assessment (IATS), Porto Alegre, RS Brazil

**Keywords:** Leprosy, Immunosuppressors, Biological therapy, Tumour necrosis factor inhibitors

## Abstract

**Background:**

Recently developed immunosuppressive drugs, especially TNF antagonists, may enhance the risk of granulomatous infections, including leprosy. We aimed to evaluate the leprosy detection rate in patients under immunosuppression due to rheumatological, dermatological and gastroenterological diseases.

**Methods:**

We performed a systematic review of the literature by searching the PubMed, EMBASE, LILACS, Web of Science and Scielo databases through 2018. No date or language restrictions were applied. We included all articles that reported the occurrence of leprosy in patients under medication-induced immunosuppression.

**Results:**

The search strategy resulted in 15,103 articles; finally, 20 articles were included, with 4 reporting longitudinal designs. The detection rate of leprosy ranged from 0.13 to 116.18 per 100,000 patients/year in the USA and Brazil, respectively. In the meta-analysis, the detection rate of cases of leprosy per 100,000 immunosuppressed patients with rheumatic diseases was 84 (detection rate = 0.00084; 95% CI = 0.0000–0.00266; *I*^*2*^ = 0%, *p* = 0.55).

**Conclusion:**

Our analysis showed that leprosy was relatively frequently detected in medication-induced immunosuppressed patients suffering from rheumatological diseases, and further studies are needed. The lack of an active search for leprosy in the included articles precluded more precise conclusions.

**Trial registration:**

This review is registered in PROSPERO with the registry number CRD42018116275.

**Supplementary Information:**

The online version contains supplementary material available at 10.1186/s12879-021-06041-7.

## Background

Biologics are the new paradigm in the treatment of autoimmune diseases [1]. They represent the most important pharmaceutical products sold in the US, as they are the treatments most frequently prescribed for inflammatory and autoimmune conditions [2]. They act on target molecules present in the pathophysiology of autoimmune diseases, including cytokines and cell surface receptors, constituting an important treatment strategy [3]. However, the target sites of immunobiological therapies also play important roles in immune homeostasis and cell cycle control. The blockage of these pathways tends to alter immune function, with a consequent increased risk of acute, latent, or chronic infections [3]. In addition, abrupt discontinuation of these therapies may lead to a paradoxical aggravation of an ongoing infection that is triggered by the establishment of an inflammatory reconstitution syndrome or worsening of the underlying autoimmune disease [3].

The most common indications for these drugs are dermatological, rheumatological and gastroenterological conditions [4]. For instance, of the 15 indications for the most commonly sold immunobiologic, adalimumab, 14 are classified as one of these conditions [5]. Biologics are increasingly being used in areas where neglected tropical diseases are common, such as India and Brazil [6–8]. Additionally, other tropical diseases, such as leprosy, still occur in those countries [9–11]. The transmission of leprosy is still a prevalent problem in many countries, with 208,619 new cases diagnosed in 2018 [10].

Leprosy is a chronic infectious granulomatous disease caused by *Mycobacterium leprae* that affects the skin and peripheral nerves [12]. Clinically, it has two main types: the tuberculoid form, represented by a strong granulomatous reaction with few bacilli, and the lepromatous form, characterized by a high degree of bacilli infiltration [13, 14]. Along the disease spectrum, there are borderline clinical forms with immunological instability and clinical characteristics of both types [15].

The natural history of leprosy is still not completely understood, and only a minority of individuals who have contact with the pathogen develop the disease [16, 17]. The proliferation of bacilli is determined by a combination of microbial, genetic and environmental factors. Clinical disease implies the failure of human defences against *M. leprae* [18]*.* Few cases have been reported in patients using immunobiological drugs.

**Fig. 1:**
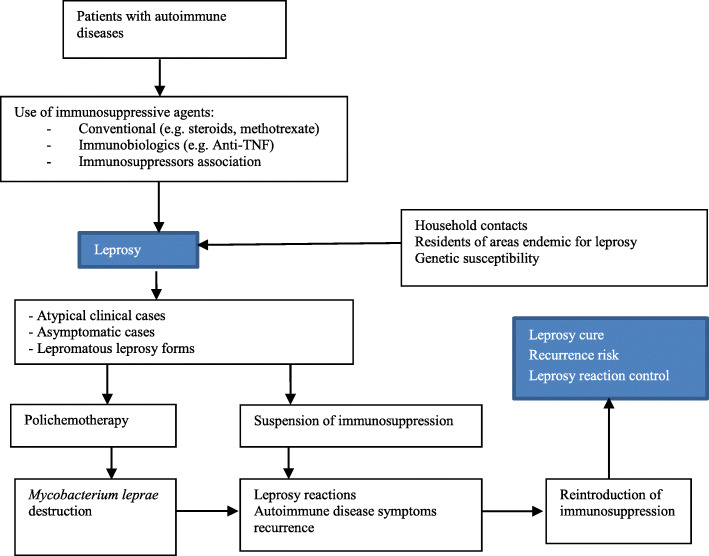
Current clinical pathway of leprosy in patients under immunosuppression due to rheumatological, dermatological and gastroenterological diseases

To reduce the risk of infections, new guidelines for the use of immunobiological therapies have been developed, with recommended patient assessments, including hepatitis B and C serology, syphilis and HIV tests, purified protein derivative skin tests or interferon gamma release assays, and chest radiography for the investigation of tuberculosis, prior to the initiation of immunobiological drug treatments. However, to date, there are no consistent data or recommendations regarding tests for other endemic diseases; new clinical pathways need to be developed for the management of leprosy in this context (Fig. 1).

To gain more insight into the magnitude of the problem, we aimed to evaluate the detection rate of leprosy in patients under immunosuppression due to rheumatological, dermatological and gastroenterological diseases. We also aimed to create a clinical profile of affected patients to describe the reported natural history of *M. leprae* infection under these conditions and the aspects of leprosy treatment.

## Methods

This review is registered in PROSPERO under the registry number CRD42018116275. On November 22nd, 2018, we comprehensively searched the PubMed, EMBASE, Scopus, Web of Science and Scielo databases. No date, language or method restrictions were applied (S1 Table 1). We included all articles that reported the occurrence of leprosy in patients under immunosuppression for dermatological, rheumatological, and gastroenterological diseases.

We excluded articles that reported immunosuppression due to other conditions, such as HIV infection, chemotherapy and organ transplantation, because of the different profiles of immunosuppression. We also excluded articles that reported leprosy cases that clearly occurred before immunosuppression and cases in which leprosy was wrongly treated as a rheumatic disease when clinical manifestations of both diseases presented considerable similarities. In addition, the analysis of follow-up treatment for leprosy in patients undergoing immunosuppressive therapy should be considered in the future; a more detailed analysis with a different search strategy is needed.

### Article selection and data extraction

After the search, all the retrieved articles were exported to EPPI-Reviewer 4 Version 4.6.4.0 (EPPI Centre, London, England), and duplicates were removed with the aid of the automatic tool in the program and subsequently manually checked. Due to the high number of references included, two groups of two independent reviewers (CG (investigator) + JB (epidemiologist) and DB (investigator) + EA (epidemiologist)) screened the titles and abstracts. Disagreements were resolved by a third reviewer (PK (investigator)).

Two independent reviewers screened the full texts and extracted the data (CG + DB and CG + AC (investigator)). A third reviewer resolved any disagreements in both steps (PK). The references of selected articles were also screened. The extracted data are presented in S2 Table 2.

### Quality assessment

Although there is considerable variability in the available methods of outcome evaluation, we opted to use the Johanna Briggs Checklist for Analytical Cross-Sectional Studies. This tool is capable of generating a homogeneous evaluation for studies in which the extraction of period prevalence data (expressed as the leprosy detection rate) is possible even when screening studies that do not necessarily include groups with different exposures. This tool evaluates the quality and the risk of bias based on 8 questions. Two independent reviewers (CG and PK) evaluated all longitudinal studies, and disagreements were resolved by consensus.

### Data analysis

An initial analysis of the included leprosy cases was performed with the aim of creating a clinical and epidemiological profile of leprosy after medication-induced immunosuppression. We searched for factors such as the type of autoimmune disease, leprosy classification (paucibacillary (PB) or multibacillary (MB)) geographical distribution of leprosy cases, leprosy treatment details and leprosy reaction management in patients.

All longitudinal studies were also separately evaluated. In this step, we extracted data regarding the new case detection rate of leprosy (a proxy for incidence and risk rates when considering immunosuppression as a risk factor), following the World Health Organization (WHO) definition [19, 20]. Articles that presented similarities in quality assessments and methodological characteristics were selected for the quantitative analysis.

### Meta-analysis

For the meta-analysis, we opted to measure the total period detection rate in selected longitudinal articles to improve the comparability of the data and to avoid analytical difficulties due to event rarity. A double arcsine transformation was applied.

We used the packages “meta”, “metafor” and “weightr” in the program R Studio for Mac (RStudio, Boston, USA). RStudio: Integrated Development for R. R Studio, Inc., Boston, MA, URL http://www.rstudio.com/) to calculate and graphically present the results.

## Results

**Fig. 2:**
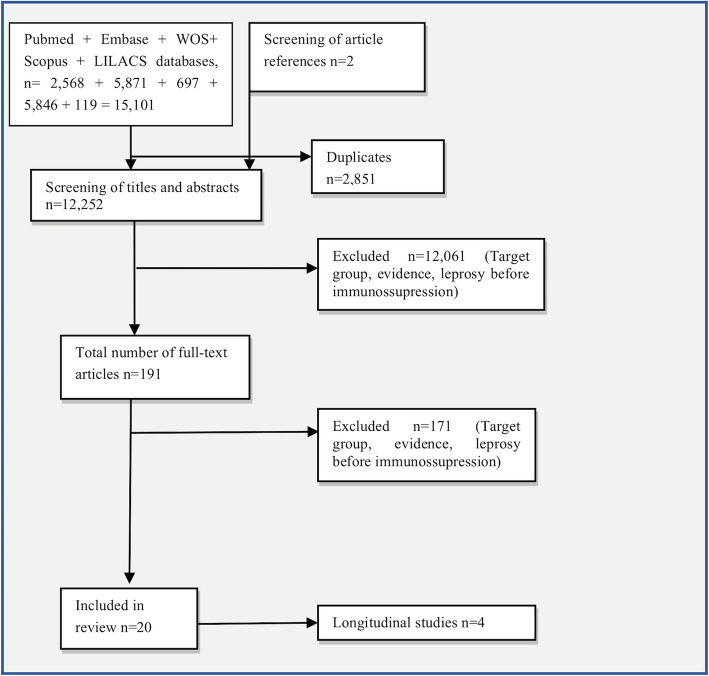
Flow diagram of article selection

The search resulted in the initial identification of 15,101 articles, and 2 additional references were added while searching the articles’ references. The application of automatic and manual duplication exclusion resulted in the exclusion of 2,851 articles. Title and abstract screening and subsequent full-text analysis resulted in the exclusion of 12,061 and 171 articles, respectively. Twenty articles were finally eligible for data extraction [12, 21–41] (Fig. 2).

Thirteen articles were case reports, and three reported leprosy case series in patients under medication-induced immunosuppression [24, 32, 37]. Four articles, including 3 observational studies [23, 31, 35] and one clinical trial [36], evaluated the detection rate of leprosy in immunosuppressed patients using longitudinal study designs. In total, all the included articles reported 24 cases of leprosy in medication-induced immunosuppressed patients.

### Clinical profile of leprosy patients

The mean age of leprosy patients who were diagnosed after immunosuppression was 48.37 years old (standard deviation (SD) = 17.01). Most reported cases were in females (57.89%), and almost all patients developed leprosy after immunosuppressive treatment for rheumatological diseases (95.24% of the reported cases). Thirteen patients had rheumatoid arthritis (1 juvenile form), 4 patients had systemic lupus erythematosus (1 juvenile form), 2 had psoriatic arthritis and 1 had ankylosing spondylitis. Only one patient had psoriasis [28], and no patient had gastroenterological disease. In three cases, the exact reason for medication-induced immunosuppression was not disclosed.

**Fig. 3:**
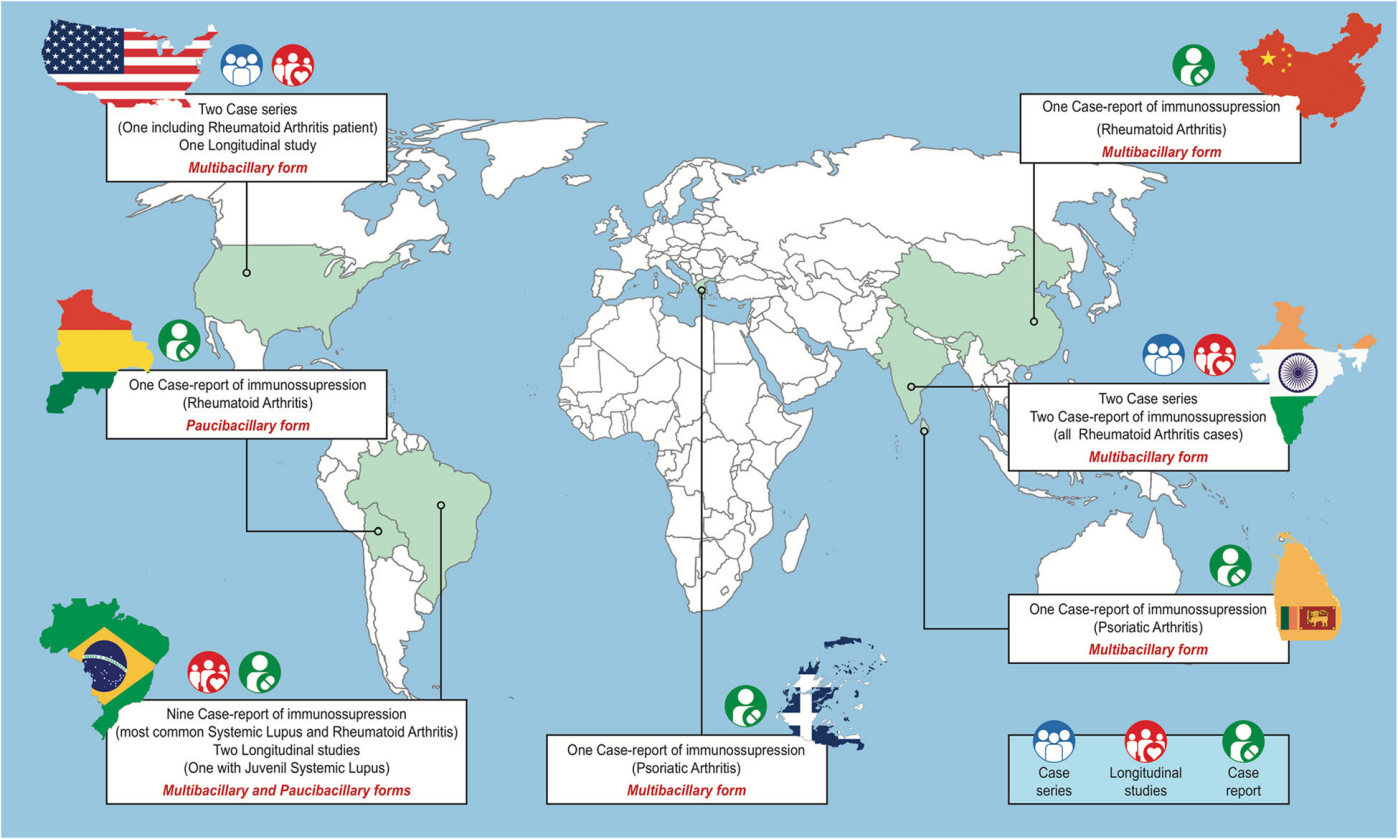
Geographical distribution of the included articles and information relevant to the clinical profile of leprosy under medication-induced immunosuppression. *Map constructed by SV using the program Adobe inDesign (2019, CA, USA)

Sixteen patients were reported to have been in hyperendemic countries at some point in their lives [42]: 11 had been in Brazil, 3 had been in India and 1 had been in Sri Lanka. One patient had been in Bolivia, and 1 patient was from China. Interestingly, 1 patient was from Greece [33], and 3 were from the USA [31, 37]. These 4 patients acquired leprosy in countries considered nonendemic for leprosy. The patient described by Lydakis C et al. in 2012 reported that her father had been diagnosed with leprosy in Greece 70 years before and had lived on Spinalonga Island, where persons with leprosy were confined [33]. Scollard DM et al. 2006 reported two cases of leprosy in the USA. One patient had never travelled outside the country, and the other patient reported having had contact with her husband, who hunted armadillos [37]. In 6 reported cases, the classification of PB leprosy was described, and in ten patients, the classification of MB leprosy was described. This information was not clear in the remaining cases. Treatment followed the WHO recommendations for the above-mentioned classifications (Fig. 3).

The mean duration of autoimmune disease in this population prior to the diagnosis of leprosy was 11.90 years (SD = 13.94). Thirteen patients were using immunobiologics alone or in combination with other immunosuppressive drugs (12 used anti-TNF drugs (7 infliximab, 3 etanercept, 1 adalimumab, and 1 not specified) and 1 used an anti-IL-6 drug (tocilizumab)). Eleven patients reported a history of steroid use for autoimmune disease control. Only three patients reported the use of only conventional immunosuppressive drugs for autoimmune disease control. Two patients used only methotrexate for the control of rheumatoid arthritis, and one used a combination of azathioprine, cyclophosphamide and mycophenolate mofetil for the control of systemic lupus erythematosus.

Immunosuppression was not terminated after the diagnosis of leprosy was made in only 2 patients [35, 37]. Eleven patients developed leprosy reactions (9 Type 1 and 2 Type 2 reactions) after the specific diagnosis. In 6 cases, the reinitiation of immunosuppression was necessary to control the leprosy reactions. In two cases, the authors clearly reported that the treatment of leprosy with the WHO-recommended polychemotherapy did not lead to an adequate response. One of these patients was treated with the PB treatment scheme (S1 Table 2).

### Diagnostic rate evaluation

Four articles with longitudinal designs reported the diagnosis rate of leprosy in patients under medication-induced immunosuppression [23, 31, 35, 36]. In total, 199,611 immunosuppressed patients were evaluated, and 4 cases of leprosy were reported in this population, resulting in a period diagnostic rate of 2 cases per 100,000 medication-induced immunosuppressed patients (pooled detection rate in the total period = 2.00 × 10^− 5^; 95% CI = 6.42 × 10^− 6^-5.51 × 10^− 5^). The detection rate of leprosy in the 4 included articles ranged from 0.13 leprosy cases per 100,000 patients/year in the USA [31] to 116.18 [23] leprosy cases per 100,000 patients/year in Brazil. However, different methodologies precluded a direct comparison among all 4 studies (Table 1).

**Table 1:** Epidemiological characteristics of the 4 included articles that presented longitudinal data

Author	Autoimmune disease	Medications	Cases (n)	Population (n)	Screening time (years)	Period detection rate/100,000	Detection rate/100,000/year
Lopes 2015 [35]	Juvenile Lupus Erythematosus	Prednisone, Hydroxychloroquine, Mycophenolate mofetil, Cyclophosphamide	1	312	32	320.50	10.02
Burmester 2016 [36]	Rheumatoid Arthritis	Tocilizumab	1	1262	2	79.20	39.62
Titton 2011 [23]	Rheumatic Diseases;	Anti-TNF and DMARDs	1	1037	0.83	96.43	116.18
Wallis RS 2005 [31]	Not clear	Infliximab	1	197,000	4	0.51	0.13
Total*	–	–	4	199,611	–	2.00	–

*n* Number of patients, *DMARDs* Disease-modifying antirheumatic drugs, * Unadjusted values

### Quality assessment

**Fig. 4:**
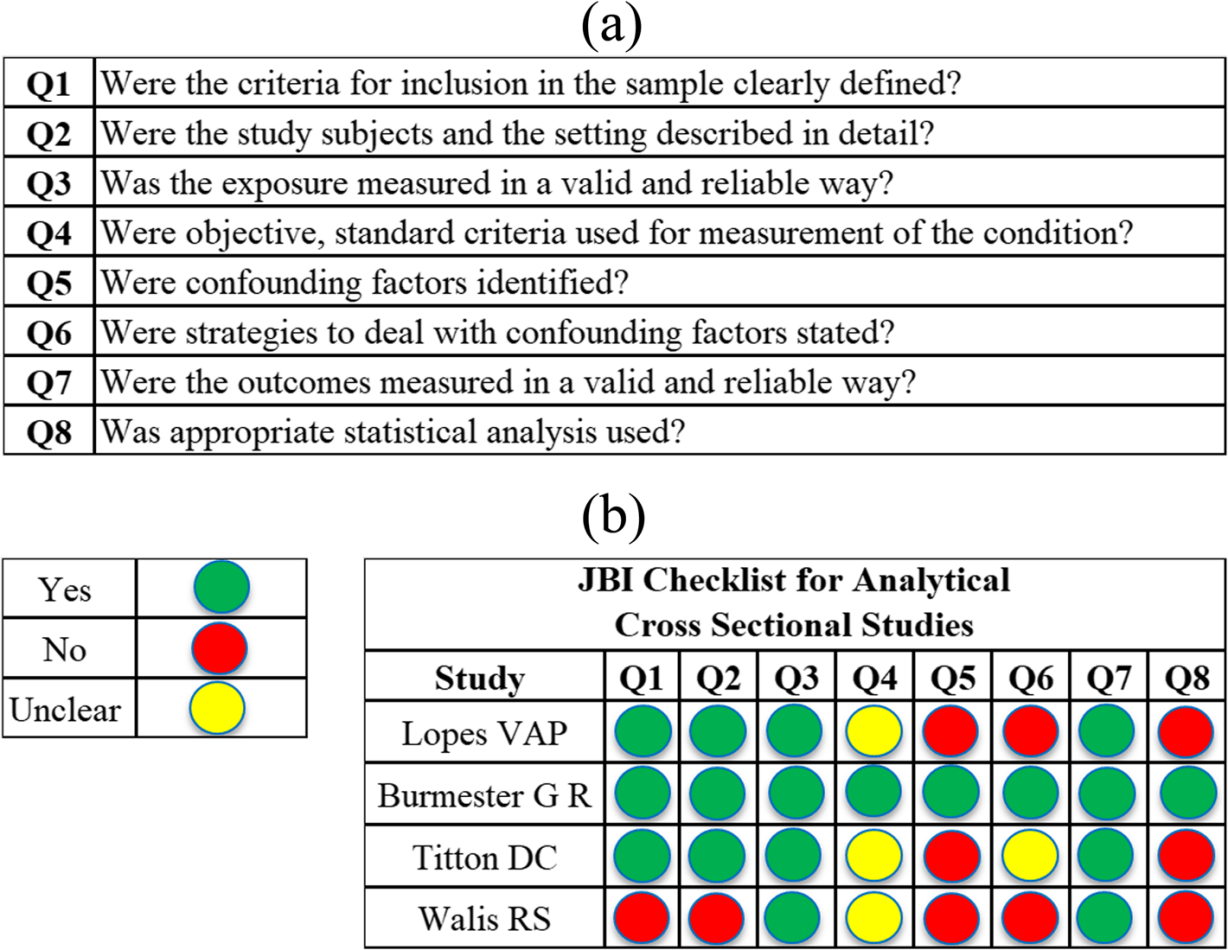
Quality assessment of the longitudinal studies. **a** The Joanna Briggs Institute (JBI) Checklist for Analytical Cross-Sectional Studies; **b** evaluation of longitudinal studies

Quality assessments were performed for the 4 longitudinal studies (Fig. 4). Only one article was scored as unclear for questions related to the inclusion criteria and the characteristics of the included subjects (Questions 1 and 2, 31]. The outcome measurement, which was the diagnosis of leprosy (Question 6), was considered adequate in all articles, as the WHO has established a clear definition of leprosy. One article was not included in the meta-analysis because the description of the included subjects was unclear [31], unlike the other remaining longitudinal studies that evaluated immunosuppressed patients suffering from rheumatological diseases.

### Meta-analysis

**Fig. 5:**
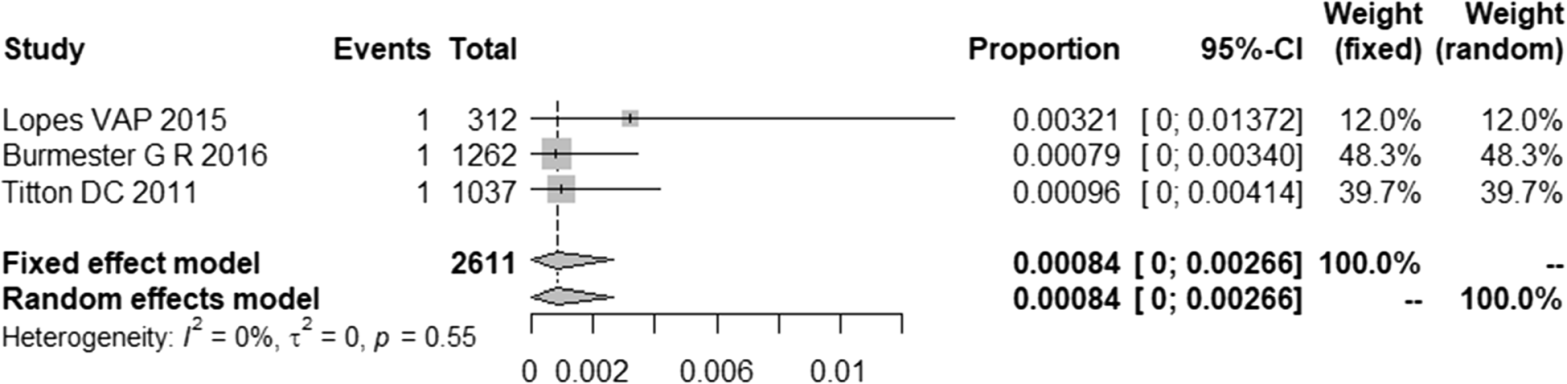
Effect sizes and confidence intervals after double arcsine transformation

The detection rate was 84 cases of leprosy per 100,000 medication-induced immunosuppressed patients who were being treated for rheumatic diseases (period detection rate = 0.00084; 95% CI = 0.0000–0.00266; *I*^*2*^ = 0%, *p* = 0.55, 23, 35, 36] (Fig. 5).

## Discussion

Leprosy is an endemic disease that reduces the quality of life in affected patients [40]. The disease affects underdeveloped regions of the world where important improvements in the quality of basic health are still needed [10]. Immunosuppression is considered an important risk factor for granulomatous infectious diseases [41].

The treatment of autoimmune diseases has undergone a substantial evolution in recent years. Previously, these conditions were treated with nonspecific medications, such as corticosteroids, methotrexate and cyclosporine [43]. Today, treatment has become personalized, and newly developed immunobiologics are becoming available. Regarding leprosy, a main concern is the targeted use of anti-TNF compounds because they are responsible for reducing the granulomatous response, an important factor for the control of mycobacterial infection [44, 45].

No evidence was found regarding the need to interrupt immunosuppression after the diagnosis of leprosy, a common action after the diagnosis of infections in immunosuppressed patients. Indeed, the reinitiation of immunosuppressive drugs was necessary in 6 patients to control reactional states after the introduction of polychemotherapy.

The leprosy detection rate in the 4 included longitudinal articles ranged from 0.13 in the USA to 116.18 leprosy cases per 100,000 patients/year in Brazil. In 2018, only 185 cases of leprosy were reported in the USA (0.06 cases per 100,000) [10]. Our meta-analysis showed a detection rate of 84 cases of leprosy per 100,000 medication-induced immunosuppressed patients suffering from rheumatological diseases. This detection rate is 30 times higher than the worldwide detection rate reported in 2018 for the general population (2.74/100,000) [10] (*p* < 0.001; 95% CI = 8.85–88.56). This detection rate was also 6 times higher than the detection rate reported in endemic countries, such as Brazil, in 2018 (13.68/100,000, 10] (*p* < 0.001; 95% CI = 3.41–10.56).

Data analyses point to a higher leprosy detection rate in patients with medication-induced immunosuppression suffering from rheumatological diseases than in the general population. However, some limitations might be considered. Initially, the small number of studies precluded the evaluation of publication bias and the consideration of confounders in a meta-regression model. It is also important to state that the variability in case samples was too low (only one case per study), and considerable variations attributable to chance may have influenced our results. Additionally, event rarity may have led to an overestimation of effect sizes [46]. Another limiting factor related to methodology is the fact that no article regarded leprosy as a primary outcome, which might have reduced the reported incidence of leprosy cases.

In the present article, our model showed that the leprosy detection rate was elevated in patients with medication-induced immunosuppression suffering from rheumatological diseases. The lack of methodological rigour and the absence of an active search for leprosy in the included articles preclude more precise conclusions. New studies considering the detection of leprosy as a primary outcome in special populations, including immunosuppressed patients, will be needed to answer the remaining questions. In addition, due to the importance of the present topic, specialists practicing in endemic countries must discuss the implementation of an active leprosy screening strategy before the initiation of immunosuppressive treatment for rheumatological diseases.

## Supplementary Information

**Additional file 1: Table S1.** Search strategy and databases accessed for the systematic review of the literature.

**Additional file 2: Table S2.** Data extracted.

## Data Availability

All data generated or analysed during this study are included in this published article and its supplementary information files.

## Abbreviations

PB: Paucibacillary
MB: Multibacillary
WHO: World Health Organization
DMARDs: Disease-modifying antirheumatic drugs
JBI: The Joanna Briggs Institute

## References

[1] Wang L, Wang F-S, Gershwin ME (2015). Human autoimmune diseases: a comprehensive update. J Intern Med.

[2] Walsh G (2018). Biopharmaceutical benchmarks 2018. Nat Biotechnol.

[3] Fernandez-Ruiz M, Meije Y, Manuel O, Akan H, Carratala J, Aguado JM, et al. ESCMID Study Group for infections in compromised hosts (ESGICH) consensus document on the safety of targeted and biological therapies: an infectious diseases perspective (introduction). Clin Microbiol Infect. 2018;24(Suppl 2):S2–s9. 10.1016/j.cmi.2018.01.029. 10.1016/j.cmi.2018.01.029 29427801

[4] Wang X, Wong SH, Wang X-S, Tang W, Liu C-Q, Niamul G, et al. Risk of tuberculosis in patients with immune-mediated diseases on biological therapies: a population-based study in a tuberculosis endemic region. Rheumatology. 2019;58(5):803–10. 10.1093/rheumatology/key364. 10.1093/rheumatology/key364 30561745

[5] Burmester GR, Gordon KB, Rosenbaum JT, Arikan D, Lau WL, Li P, et al. Long-term safety of Adalimumab in 29,967 adult patients from global clinical trials across multiple indications: an updated analysis. Adv Ther. 2020;37(1):364–80. 10.1007/s12325-019-01145-8. 10.1007/s12325-019-01145-8 PMC6979455 31748904

[6] Yonekura CL, Oliveira RDR, Titton DC, Ranza R, Ranzolin A, Hayata AL, et al. Incidence of tuberculosis among patients with rheumatoid arthritis using TNF blockers in Brazil: data from the Brazilian Registry of Biological Therapies in Rheumatic Diseases (Registro Brasileiro de Monitoração de Terapias Biológicas–BIOBADABRASIL). Rev Br Reumatol. 2017;57:477–83. 10.1016/j.rbre.2017.05.005 28739353

[7] Jayaraman K (2010). India’s Cipla sets sights on Avastin, Herceptin and Enbrel. Nat Biotechnol.

[8] Saberwal G (2013). Giving voice to India’s entrepreneurs. Nat Biotechnol.

[9] Cardenas VM, Orloff MS, Kaminaga J, Cardenas IC, Brown J, Hainline-Williams S, et al. Tuberculosis and leprosy infections in the Marshallese population of Arkansas, USA. Lepr Rev. 2016;87(1):109–12. 10.47276/lr.87.1.109. 27255065

[10] World Health Organization (2019). Global leprosy update, 2018: moving towards a leprosy-free world. Weekly epidemiological record.

[11] Valentín DC, Candelario N, Carrasquillo OY, Figueroa L, Sánchez JL (2017). Leprosy in Puerto Rico: insight into the new millennia. Int J Dermatol.

[12] Lopes RV, Ohashi CB, Cavaleiro LH, Cruz RBP, da Veiga RRG, Miranda MFR, et al. Development of leprosy in a patient with ankylosing spondylitis during the infliximab treatment: reactivation of a latent infection? Clin Rheumatol. 2009;28(5):615–7. 10.1007/s10067-009-1140-0. 10.1007/s10067-009-1140-0 19259757

[13] Desikan K, Job C (1968). A review of postmortem findings in 37 cases of leprosy. Int J Leprosy.

[14] Ridley D (1974). Histological classification and the immunological spectrum of leprosy. Bull World Health Organ.

[15] Frade MAC, de Paula NA, Gomes CM, Vernal S, Bernardes Filho F, Lugão HB, et al. Unexpectedly high leprosy seroprevalence detected using a random surveillance strategy in midwestern Brazil: a comparison of ELISA and a rapid diagnostic test. PLoS Negl Trop Dis. 2017;11(2):e0005375. 10.1371/journal.pntd.0005375. 10.1371/journal.pntd.0005375 PMC5358972 28231244

[16] Naafs B (2018). World leprosy day 2018: how forward respecting the past?. Indian J Med Res.

[17] Araujo S, Freitas LO, Goulart LR, Goulart IMB (2016). Molecular evidence for the aerial route of infection of mycobacterium leprae and the role of asymptomatic carriers in the persistence of leprosy. Clin Infect Dis.

[18] Casanova J-L, Abel L (2002). Genetic dissection of immunity to mycobacteria: the human model. Annu Rev Immunol.

[19] World Health Organization (2018). Guidelines for the diagnosis, treatment and prevention of leprosy.

[20] Organization WH (2019). Global leprosy strategy 2016–2020: accelerating towards a leprosy-free world.

[21] Lluch P, Urruticoechea A, Lluch J, Moll MC, Matos M, Benet JM, et al. Development of leprosy in a patient with rheumatoid arthritis during treatment with etanercept: a case report. In: Seminars in arthritis and rheumatism: 2012: Elsevier; 2012. p. 127–30. 10.1016/j.semarthrit.2012.03.003 22542278

[22] Freitas DS, Machado N, Andrigueti FV, Reis Neto ET, Pinheiro MM (2010). Lepromatous leprosy associated with the use of anti-TNF α therapy: case report. Rev Bras Reumatol.

[23] Titton DC, Silveira IG, Louzada-Junior P, Hayata AL, Carvalho HMS, Ranza R, et al. Brazilian biologic registry: BiobadaBrasil implementation process and preliminary results. Rev Bras Reumatol. 2011;51(2):152–60. 10.1590/S0482-50042011000200005. 21584421

[24] Ribeiro F, Gomez V, Albuquerque E, Klumb E, Shoenfeld Y (2015). Lupus and leprosy: beyond the coincidence. Immunol Res.

[25] Kaur M, Grindulis K, Maheshwari M, Ellis C, Bhat J, Tan C (2007). Delayed diagnosis of leprosy due to presentation with a rheumatoid-like polyarthropathy. Clin Exp Dermatol.

[26] Henriques CC, Lopéz B, Mestre T, Grima B, Panarra A, Riso N (2012). Leprosy and rheumatoid arthritis: consequence or association?. Case Rep.

[27] Oliveira C, Carreño A, Francesconi F, Peixoto I, Campos J, de Fátima Maroja M, et al. Hansen disease after etanercept treatment. J Am Acad Dermatol. 2013;68(4).

[28] Santos EBE, Brito G, Fernades A, Dias J (2013). Posters and oral communications- leprosy tuberculoid associated with psoriasis treated with immunobiological: report of a clinical case: P 198. J Eur Acad Dermatol Venereol.

[29] Sindhu B, Sathaiah, Kavitha, Rani S, Srinivas, Kumar U, et al. Atypical presentations of Hansen’s disease. Indian J Lepr. 2015;87(3):222.

[30] Sobanko JF, Freeman AF, Palmore TN, Mendoza D, Richard Lee CC, Cowen EW (2009). A Sri Lankan woman with rheumatoid arthritis and anesthetic plaques. J Am Acad Dermatol.

[31] Wallis RS, Broder M, Wong J, Lee A, Hoq L (2005). Reactivation of latent granulomatous infections by infliximab. Clin Infect Dis.

[32] Salvi S, Chopra A (2013). Leprosy in a rheumatology setting: a challenging mimic to expose. Clin Rheumatol.

[33] Lydakis C, Ioannidou D, Koumpa I, Giannikaki E, Thalassinos E, Krasoudaki E, et al. Development of lepromatous leprosy following etanercept treatment for arthritis. Clin Rheumatol. 2012;31(2):395–8. 10.1007/s10067-011-1903-2. 10.1007/s10067-011-1903-2 22170033

[34] Teixeira FM, Vasconcelos LMF, Rola CD, de Almeida TLP, Valença JT, Nagao-Dias AT (2011). Secondary leprosy infection in a patient with psoriasis during treatment with infliximab. J Clin Rheumatol.

[35] Lopes V, Lourenco D, Guariento A, Trindade M, Avancini J, Silva C (2015). Borderline tuberculoid leprosy in childhood onset systemic lupus erythematosus patient. Lupus.

[36] Burmester GR, Rubbert-Roth A, Cantagrel A, Hall S, Leszczynski P, Feldman D, et al. Efficacy and safety of subcutaneous tocilizumab versus intravenous tocilizumab in combination with traditional DMARDs in patients with RA at week 97 (SUMMACTA). Ann Rheum Dis. 2016;75(1):68–74. 10.1136/annrheumdis-2015-207281. 10.1136/annrheumdis-2015-207281 PMC4717437 26056119

[37] Scollard D, Joyce M, Gillis T (2006). Development of leprosy and type 1 leprosy reactions after treatment with infliximab: a report of 2 cases. Clin Infect Dis.

[38] Agrawal S, Sharma A (2007). Dual mycobacterial infection in the setting of leflunomide treatment for rheumatoid arthritis. Ann Rheum Dis.

[39] Antônio JR, Soubhia RMC, Paschoal VDA, Amarante CF, Travolo ARF (2013). Biological agents: investigation into leprosy and other infectious diseases before indication. An Bras Dermatol.

[40] Limeira OM, Gomes CM, Morais OO, Cesetti MV, Alvarez RRA (2013). Active search for leprosy cases in Midwestern Brazil: a serological evaluation of asymptomatic household contacts before and after prophylaxis with bacillus Calmette-Guérin. Rev Inst Med Trop Sao Paulo.

[41] Kurizky P, Gomes C, Cesetti M, Martins G, Regattieri N, Marianelli F, et al. Cross-sectional screening study for Leishmania DNA and antibodies in biologic-treated patients with psoriasis living in an area endemic for leishmaniasis. Br J Dermatol. 2019;181(6):1337–9. 10.1111/bjd.18262. 10.1111/bjd.18262 31260088

[42] Penna MLF, Penna GO (2012). Leprosy frequency in the world, 1999-2010. Mem Inst Oswaldo Cruz.

[43] Kremer JM (2016). The Corrona US registry of rheumatic and autoimmune diseases. Clin Exp Rheumatol.

[44] Aarestrup FM, Sampaio EP, de Moraes MO, Albuquerque E, Castro A, Sarno EN (2000). Experimental mycobacterium leprae infection in BALB/c mice: effect of BCG administration on TNF-a production and granuloma development. Int J Lepr Other Mycobact Dis.

[45] Hagge DA, Saunders BM, Ebenezer GJ, Ray NA, Marks VT, Britton WJ, et al. Lymphotoxin-α and TNF have essential but independent roles in the evolution of the granulomatous response in experimental leprosy. Am J Pathol. 2009;174(4):1379–89. 10.2353/ajpath.2009.080550. 10.2353/ajpath.2009.080550 PMC2671369 19246648

[46] Israel H, Richter RR (2011). A guide to understanding meta-analysis. J Orthop Sports Phys Ther.

