# Supplementary material for: Leprosy detection rate in patients under immunosuppression for the treatment of dermatological, rheumatological, and gastroenterological diseases: a systematic review of the literature and meta-analysis
Source: BMC Infect Dis. 2021 Apr 13;21:347. doi: 10.1186/s12879-021-06041-7 (PMC8045377; doi:10.1186/s12879-021-06041-7)
Supplement: Supplementary file 1 — Additional file 1: Table S1. Search strategy and databases accessed for the systematic review of the literature. [file 12879_2021_6041_MOESM1_ESM.docx]

Supporting table 1. Search strategy and databases accessed for the systematic review of the literature.

| Accessed databases | Search strategy |
| --- | --- |
| PUBMED | (((((leprae) OR leprosy) OR lepromatosis) OR hansen*)) AND (((corticoid* OR steroid* OR methotrexate OR leflunomide OR cyclosporine OR infliximab OR adalimumab OR etanercept OR golimumab OR certolizumab* OR secukinumab OR ustekinumab OR apremilast OR abatacept OR azathioprine OR mycophenol* OR rituximab OR sulfasalazine OR tocilizumab OR tofacitinib OR) OR immunos*)) |
| LILACS | (leprae OR leprosy OR lepromatosis OR hansen*) AND (corticoid* OR steroid* OR methotrexate OR leflunomide OR cyclosporine OR infliximab OR adalimumab OR etanercept OR golimumab OR certolizumab* OR secukinumab OR ustekinumab OR apremilast OR abatacept OR azathioprine OR mycophenol* OR rituximab OR sulfasalazine OR tocilizumab OR tofacitinib OR immunos*) |
| SCOPUS | ( ( ALL ( immunosuppression ) ) OR ( ( ALL ( corticoid* ) OR ALL ( steroid* ) OR ALL ( methotrexate ) OR ALL ( leflunomide ) OR ALL ( cyclosporine ) OR ALL ( infliximab ) OR ALL ( adalimumab ) OR ALL ( etanercept ) OR ALL ( golimumab ) OR ALL ( certolizumab* ) OR ALL ( secukinumab ) OR ALL ( ustekinumab ) OR ALL ( apremilast ) OR ALL ( abatacept ) OR ALL ( azathioprine ) OR ALL ( mycophenol* ) OR ALL ( rituximab ) OR ALL ( sulfasalazine ) OR ALL ( tocilizumab ) OR ALL ( tofacitinib ) ) ) ) AND ( ALL ( leprosy ) ) |
| Web of Science | #4 AND #3  *Índices=SCI-EXPANDED, SSCI, A&HCI, CPCI-S, CPCI-SSH, BKCI-S, BKCI-SSH, ESCI, CCR-EXPANDED, IC Tempo estipulado=Todos os anos*  **#4** #2 OR #1  *Índices=SCI-EXPANDED, SSCI, A&HCI, CPCI-S, CPCI-SSH, BKCI-S, BKCI-SSH, ESCI, CCR-EXPANDED, IC Tempo estipulado = Todos os anos*  **#3** TS=(leprae* OR leprosy OR lepromatosis OR hansen*)  *Índices=SCI-EXPANDED, SSCI, A&HCI, CPCI-S, CPCI-SSH, BKCI-S, BKCI-SSH, ESCI, CCR-EXPANDED, IC Tempo estipulado = Todos os anos*  **#2** TS=(immunos*)  *Índices=SCI-EXPANDED, SSCI,A&HCI, CPCI-S, CPCI-SSH, BKCI-S, BKCI-SSH, ESCI, CCR-EXPANDED, IC Tempo estipulado = Todos os anos*  **#1** TS=(corticoid* OR steroid* OR methotrexate OR leflunomide OR cyclosporine OR infliximab OR adalimumab OR etanercept OR golimumab OR certolizumab* OR secukinumab OR ustekinumab OR apremilast OR abatacept OR azathioprine OR mycophenol* OR rituximab OR sulfasalazine OR tocilizumab OR tofacitinib)  *Índices=SCI-EXPANDED, SSCI,A&HCI, CPCI-S, CPCI-SSH, BKCI-S, BKCI-SSH, ESCI, CCR-EXPANDED, IC Tempo estipulado = Todos os anos* |
| EMBASE | **#5** #3 AND #4  **#4** #1 OR #2  **#3** leprae OR ‘leprosy’/exp OR leprosy OR lepromatosis OR hansen*  **#2** immunos*  **#1** corticoid* OR steroid* OR ‘methotrexate’/exp OR methotrexate OR ‘leflunomide’/exp OR leflunomide OR ‘cyclosporine’/exp OR cyclosporine OR ‘infliximab’/exp OR infliximab OR ‘adalimumab’/exp OR adalimumab OR ’etanercept’/exp OR etanercept OR ‘golimumab’/exp OR golimumab OR certolizumab* OR ‘secukinumab’/exp OR secukinumab OR ‘ustekinumab’/exp OR ustekinumab OR ‘apremilast’/exp OR apremilast OR ‘abatacept’/exp OR abatacept OR ‘azathioprine’/exp OR azathioprine OR mycophenol* OR ‘rituximab’/exp OR rituximab OR ‘sulfasalazine’/exp OR sulfasalazine OR ‘tocilizumab’/exp OR tocilizumab OR ‘tofacitinib’/exp OR tofacitinib |
